# Effect of *CYP3A4*, *CYP3A5*, *MDR1* and *POR* Genetic Polymorphisms in Immunosuppressive Treatment in Chilean Kidney Transplanted Patients

**DOI:** 10.3389/fphar.2021.674117

**Published:** 2021-12-06

**Authors:** Stephania Contreras-Castillo, Anita Plaza, Jana Stojanova, Gustavo Navarro, Rodolfo Carmona, Fernando Corvalán, Leslie Cerpa, Christopher Sandoval, Daniel Muñoz, Marina Leiva, Luis E. Castañeda, Nayaret Farias, Carolina Alvarez, Gabriel Llull, Sergio Mezzano, Leopoldo Ardiles, Nelson Varela, María S. Rodríguez, Claudio Flores, Juan Pablo Cayún, Paola Krall, Luis A. Quiñones

**Affiliations:** ^1^ Laboratory of Chemical Carcinogenesis and Pharmacogenetics (CQF), Department of Basic and Clinical Oncology, Faculty of Medicine, University of Chile, Santiago, Chile; ^2^ Laboratory of Nephrology, Universidad Austral de Chile, Valdivia, Chile; ^3^ Interdisciplinary Centre for Health Studies (CIESAL), Universidad de Valparaíso, Valparaíso, Chile; ^4^ Department of Clinical Pharmacology and Toxicology, St. Vincent’s Hospital, Sydney, NSW, Australia; ^5^ Latin American Network for Implementation and Validation of Clinical Pharmacogenomics Guidelines (RELIVAF-CYTED), Madrid, Spain; ^6^ Pharmacy Institute, Faculty of Sciences, Universidad Austral de Chile, Valdivia, Chile; ^7^ Program of Human Genetics, Institute of Biomedical Sciences, Faculty of Medicine, University of Chile, Santiago, Chile; ^8^ Transplantation Unit, San Juan de Dios Hospital, Santiago, Chile; ^9^ Departament of Pediatrics and Child Surgery, Faculty of Medicine, University of Chile, Santiago, Chile

**Keywords:** polymorphisms, pharmacogenetics, kidney transplant, cyclosporine, tacrolimus

## Abstract

Cyclosporine (CsA) and tacrolimus (TAC) are immunosuppressant drugs characterized by a narrow therapeutic range and high pharmacokinetic variability. The effect of polymorphisms in genes related to the metabolism and transport of these drugs, namely *CYP3A4*, *CYP3A5*, *MDR1* and *POR* genes, has been evaluated in diverse populations. However, the impact of these polymorphisms on drug disposition is not well established in Latin American populations. Using *TaqMan*® probes, we determined the allelic frequency of seven variants in *CYP3A4*, *CYP3A5*, *MDR1* and *POR* in 139 Chilean renal transplant recipients, of which 89 were treated with CsA and 50 with TAC. We tested associations between variants and trough and/or 2-hour concentrations, normalized by dose (C_0_/D and C_2_/D) at specific time points post-transplant. We found that CYP3A5*3/*3 carriers required lower doses of TAC. In TAC treated patients, most CYP3A5*3/*3 carriers presented higher C_0_/D and a high proportion of patients with C_0_ levels outside the therapeutic range relative to other genotypes. These results reinforce the value of considering *CYP3A5* genotypes alongside therapeutic drug monitoring for TAC treated Chilean kidney recipients.

## Introduction

Chronic kidney disease is a global public health problem which in 2017 constituted the second and fifth cause of death in Central and Andean Latin America, respectively (Bikbov et al., 2020). These patients require dialysis or kidney transplant as renal replacement therapies. Patients that undergo transplantation require multimodal immunosuppression where a calcineurin inhibitor, either cyclosporine (CsA) or tacrolimus (TAC), is the mainstay of regimens. These drugs exhibit a narrow therapeutic index: overexposure may result in outcomes such as viral infections, nephrotoxicity and post-transplant diabetes, and underexposure puts patients at risk of allograft rejection (Cotovio et al., 2013; Egeland et al., 2017). Blood concentration levels of calcineurin inhibitors exhibit high inter- and intra-individual variability and associate with clinical outcomes (Webster et al., 2005; Rodrigo et al., 2016). Consequently, dosing is individualized based on measured blood concentrations, or therapeutic drug monitoring.

CsA and TAC are metabolized by the cytochrome P450 enzyme system, primarily the CYP3A4 and CYP3A5 isoforms. Their absorption is influenced by the P-glycoprotein transporter, ABCB1/MDR1. CYP3A4 and CYP3A5 activities are modulated by cytochrome P450 oxidoreductase enzyme, POR (Jiang et al., 2008; Jonge et al., 2011). The genes of these proteins harbor common genetic polymorphisms that are known to influence CsA and TAC disposition, and to affect clinical outcomes (Hesselink et al., 2014; Traynor et al., 2015; Sun et al., 2017; Gelder et al., 2020).

The CYP3A5*3 (rs776746, c.219-273-1A > G) polymorphism can cause alternative splicing that generates a truncated enzyme with reduced activity. The polymorphic allele (G) is more common than the wild type allele (*A*) in most populations (Kuehl et al., 2001). The impact of this polymorphism on TAC disposition is well established, as evidenced by its inclusion in an international dosing guideline from the Clinical Pharmacogenetics Implementation Consortium (CPIC) (Birdwell et al., 2015). CsA is less significantly affected, likely due to different relative affinities of CYP3A5 for TAC and CsA. CYP3A4*1B (rs2740574, c.−392A > G) is purported to cause increased enzymatic activity (Amirimani et al., 2003). The gene encoding P-glycoprotein, *MDR1*, contains several common polymorphisms, however, the impact of individual polymorphisms or common haplotypes has exhibited divergent results (Staatz et al., 2010). POR*28 C > T at nucleotide 1508 (rs1057868) has exhibited reduced CYP3A5 activity (Elens et al., 2013).

We investigated the association of common polymorphisms in *CYP3A4*, *CYP3A5*, *MDR1* and *POR* genes with blood concentrations and dose requirements in kidney transplant recipients treated with CsA or TAC from two hospitals in Chile. While the impact of these polymorphisms has been evaluated, the populations studied have not been from the Latin American region. Our work adds further support to the existing body of literature and is the first report in a particular admixed Latin American population. In addition, data from observational work adds support for genotyping relevant polymorphisms in these patients in order to more quickly achieve levels within a target range, and keep within range over the course of prolonged treatment.

## Materials and Methods

### Patients and Study Design

We performed two observational studies involving transplant units at two hospitals in Chile, where 85–90% of the transplantations were performed with deceased donors. The first study was of a retrospective cohort design and involved adults treated with CsA, transplanted at San Juan de Dios Hospital (HSJD), Santiago, between 2002 and 2013 (*n* = 89, CsA cohort). Clinical data for this cohort were collected at weeks 1, 2 and 4 post-transplant. The second study was of a cross-sectional design and involved adults treated with TAC, transplanted at Valdivia Base Hospital (HBVAL) between 2003 and 2018 (*n* = 50, TAC cohort). Data for this cohort were collected at a single time point, 6 months to 14 years post-transplant.

Inclusion criteria for both studies were kidney transplant, CsA or TAC as the primary immunosuppressant. All TAC patients used diltiazem as a tacrolimus-sparing agent. Patients receiving an alternative primary immunosuppressant after 3 months, or receiving drugs that inhibit or induce CYP3A5 significantly, were excluded. CsA and TAC doses were routinely monitored and adjusted based on C_0_ and/or C_2_ levels to achieve the respective therapeutic range (CsA C_2_ 180–250 ng/ml: TAC C_0_ 5–10 ng/ml). Levels were monitored beyond week 1 post-transplant to ensure steady state had been reached.

### Ethics

Ethical approval was obtained from the corresponding Institutional Review Board (Faculty of Medicine of University of Chile and Health Service for HSJD, and the hospital committee and Health Service for HBVAL). This study was performed according to the Declaration of Helsinki, Good Clinical Practice and Chilean Legislation (laws 20.120, 20.584 and 19.628). All patients signed an informed consent form prior to providing a sample for genotyping. Clinical data collection, including blood concentration levels and doses, was retrospective.

### Drug Measurement and Quantification

CsA and TAC blood levels were determined by a homogeneous immunoassay at both HSJD and HBVAL (Cobas E-411, Roche Diagnostics). Blood samples were obtained pre-dose for CsA or TAC (C_0_) and 2 h after administration for CsA (C_2_). CsA and TAC levels were registered and normalized by dose (C_0_/D) to perform analyses between genotypes.

### Genotype Analyses

Blood samples were collected between 2014 and 2018. For genotyping, 3–5 ml of venous blood was centrifuged for 25 min at 2500xg and 10°C to obtain the buffy coat. DNA was isolated from the peripheral blood mononuclear cells using the High Pure PCR Template Preparation kit (Roche® Diagnostics Gmbh, Mannheim, Germany) or the Whole Blood Genomic DNA Purification kit (ThermoFisher®). Purified DNA was stored at −20ºC until use. Commercial *TaqMan*® probes (ThermoFisher®) were used to determine seven polymorphisms. The thermocyclers used were Stratagene mx3000p (Agilent®) or RotorGeneQ (Qiagen®) for HSJD and HBVAL, respectively. We randomly selected 40% of the samples to validate genotype results obtained by the TaqMan assay. We used direct sequencing and/or PCR-RFLP for validation and concordance between assays was 100%. Confirmed samples representing different genotypes (homozygous reference allele, heterozygous, homozygous variant allele) were used as internal controls in each assay. Each assay contained four controls: one sample representing each genotype as positive controls (with the exception of very rare genotypes), and one negative sample with nuclease-free pure water to volume.

### Statistical Analyses

Analyses were performed using R version 4.02 (R studio version March 1, 1056). C_0_ and C_2_ levels were adjusted by dose (C_0_/D and C_2_/D), and dose was normalized by weight. Normality and homoscedasticity were tested for these variables using Shapiro-Wilks and Levene tests, respectively. To meet normality assumptions, square-root transformations were applied to C_0_/D and C_2_/D, and log10 transformation was applied to TAC-D.

Repeated measures ANOVA was performed to evaluate the differences between genotypes along 1, 2 and 4 weeks after transplantation on continuous variables (C_0_/D, Dose and eGFR). Pairwise comparisons between genotypes and weeks were analyzed by t-tests corrected by Bonferroni adjustment. TAC-D was compared between genotypes by ANOVA, and pairwise comparisons between genotypes were analyzed by t-tests corrected by Bonferroni adjustment. We also compared TAC-D between G-carriers (G/G + A/G) and A/A *CYP3A5* genotypes, and between A-carriers (A/A + A/G) and G/G *CYP3A5* genotypes using ANOVA. We also compared TAC-D adjusted by weight between G-carriers and A/A *CYP3A5* genotypes, and between A-carriers and G/G *CYP3A5* genotypes using Kruskal-Wallis tests.

Differences in proportion of patients in the therapeutic range between genotype groups was performed using the Chi-square test or Fisher test. Estimated Glomerular Filtration (eGFR) was calculated using weight, self-reported ethnicity and serum creatinine as variables (“transplantr” package version 2.0). Statistical evaluations were considered in the context of the functional consequence of each polymorphism (increased activity expected, decreased activity expected or controversial consequence). For all analyses, two-sided p-values ≤ 0.05 were considered significant.

## Results

### Demographic Characteristics of Patients

We studied 139 adult Chilean kidney transplant patients receiving CsA (64%) or TAC (36%). Demographic and clinical data are shown in Table 1. Most patients had undergone their first transplant (97.8% for the CsA-cohort and 91.1% for the TAC-cohort). A majority received organs from deceased donors (98.9% for CsA and 86.5% for TAC). The etiology of renal failure was diverse, and for most patients it was unknown or unregistered.

**TABLE 1:** Baseline characteristics of patients.

	Total (*n* = 139)
**Cohort**	
HBVAL	50 (36.0%)
HSJD	89 (64.0%)
**Sex**	
Female	67 (48.2%)
Male	72 (51.8%)
**Age** (years)	
Mean (SD)	41.8 (14.0)
Median [Min, Max]	42.0 [11.0–71.0]
**Weight** (Kg)	
Mean (SD)	65.1 (11.6)
Median [Min, Max]	65.5 [31.0, 91.0]
**Height** (mt)	
Mean (SD)	1.62 (0.100)
Median [Min, Max]	1.63 [1.30, 1.86]
**BMI** (Kg/mt2)	
Mean (SD)	24.6 (3.43)
Median [Min, Max]	24.1 [18.3, 34.2]
**Cold ischemia** (hr)	
Mean (SD)	20.1 (6.19)
Median [Min, Max]	21.3 [1.00, 33.0]

HBVAL: hospital base valdivia; HSJD: Hospital San Juan de Dios; BMI: body mass index.

All patients were genotyped for CYP3A4*1B, CYP3A5*3, CYP3A4*22, MDR1 1236 C > T, MDR1 2677 A > T, MDR1 3435 C > T and POR*28 polymorphisms. Genotype frequencies are shown in Table 2. None of the genotypes exhibited deviation from Hardy-Weinberg equilibrium.

**TABLE 2:** Genotype frequencies of patients.

	Total (*n* = 139)
CYP3A4*1B rs2740574–392A > G	
AA	115 (84%)
AG	22 (16%)
GG	0 (0%)
CYP3A5*3 rs776746 6986A > G	
AA	10 (7.2%)
AG	48 (35%)
GG	81 (58%)
CYP3A4*22 rs35599367 C191T	
CC	133 (96%)
CT	5 (3.6%)
TT	0 (0%)
*MDR1* 3435 rs1045642 C3435T	
CC	46 (33.1%)
CT	65 (46.8%)
TT	28 (20.1%)
*MDR1* 1236 rs1128503 C1236T	
CC	32 (23.0%)
CT	71 (51.1%)
TT	36 (25.9%)
*MDR1* 2677 rs2032582 C2677T	
CC	88 (64%)
CT	38 (8.7%)
TT	12 (28%)
POR*28 rs1057868 1508C > T	
CC	72 (53%)
CT	53 (39%)
TT	11 (8.1%)

### Genetic Association Between Polymorphisms and Drug Levels

**FIGURE 1:**
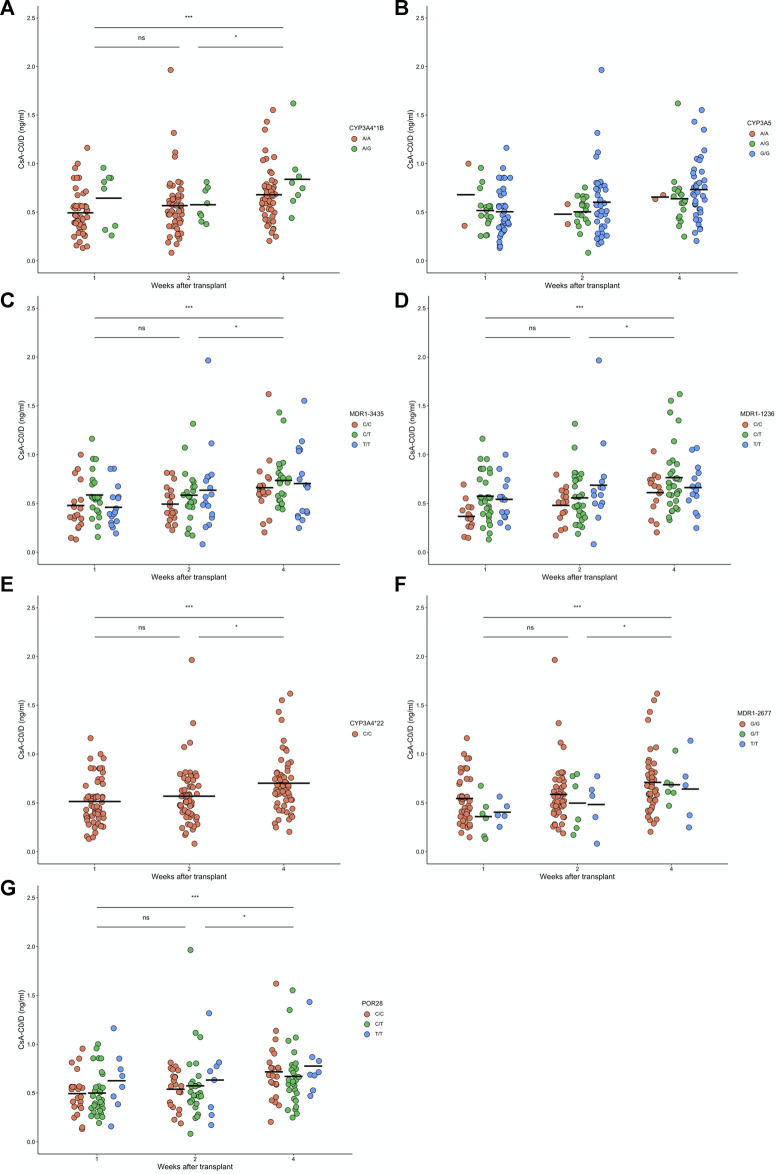
Levels of cyclosporine (CsA) normalized by dose before dose administration (CsA-C0/D) after 1, 2 and 4 weeks of renal transplant for 139 patients with different **(A)** CYP3A4*1B, **(B)** CYP3A5, **(C)** MDR1-3435, **(D)** MDR1-1236, **(E)** CYP3A4*22, **(F)** MDR1-2677, and **(G)** POR28 genotypes. For CsA-C0/D, non-differences were found between genotypes, but significance differences were found along time after transplantation (see Supplementary Table S1). Paired week comparisons using Bonferroni-adjusted t- tests are indicated as non-significant (ns: *p* > 0.05) or significant (*p < 0.05, **p < 0.01, ***p < 0.001, and ****p < 0.0001).

**FIGURE 2:**
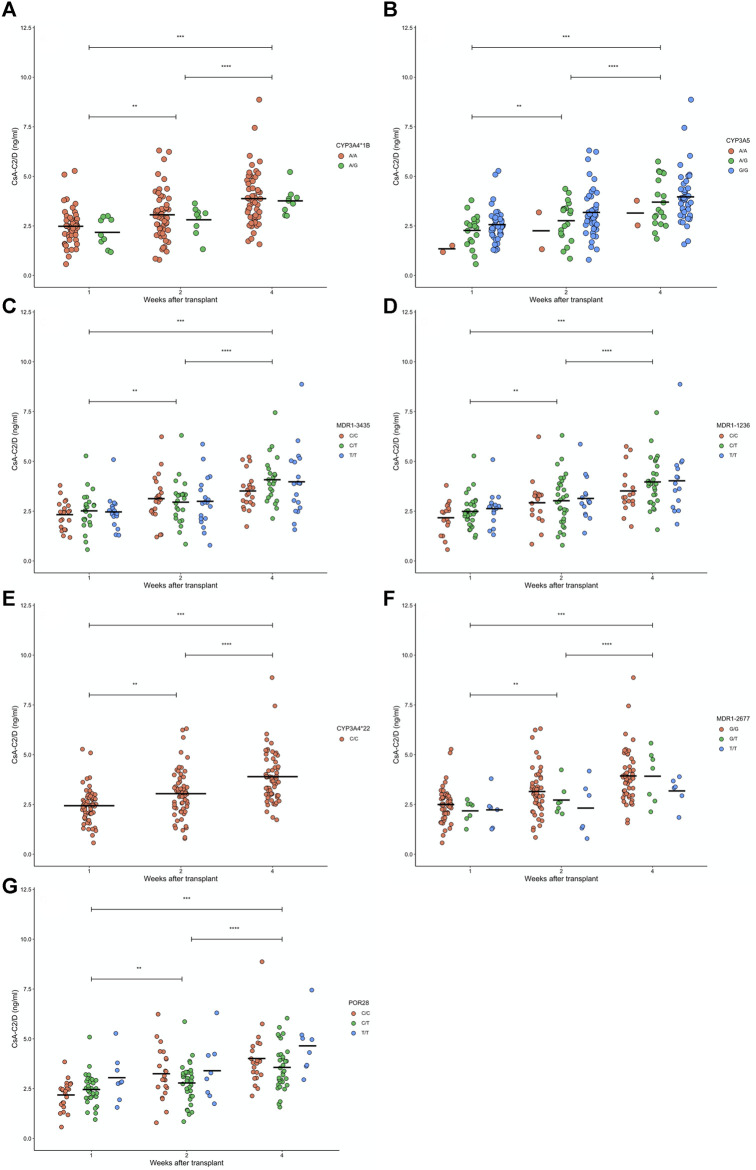
Levels of cyclosporine (CsA) normalized by dose after 2 h of administration (CsA-C0/D) after 1, 2 and 4 weeks of renal transplant for 139 patients with different **(A)** CYP3A4*1B, **(B)** CYP3A5, **(C)** MDR1-3435, **(D)** MDR1-1236, **(E)** CYP3A4*22, **(F)** MDR1-2677, and **(G)** POR28 genotypes. For CsA-C0/D, non-differences were found between genotypes, but significance differences were found along time after transplantation (see Supplementary Table S1). Paired week comparisons using Bonferroni-adjusted t- tests are indicated as non-significant (ns: *p* > 0.05) or significant (*p < 0.05, **p < 0.01, ***p < 0.001, and ****p < 0.0001).

We tested associations between polymorphisms and drug levels normalized by dose. Data from CsA treated patients were obtained at week 1, 2 and 4 after transplantation. We tested associations between genotypes and C_0_/D (Figure 1) and C_2_/D (Figure 2), at the different weeks of follow-up. We also tested combinations of *CYP3A* polymorphisms for associations, according to previous reports (Staatz et al., 2010; Okubo et al., 2013). We defined two groups: 1) low enzymatic activity: *CYP3A4* (*1A/*1A), *CYP3A4* (*22/*22), *CYP3A5* (*3/*3); and 2) intermediate or high enzymatic activity: *CYP3A4* (*1A/*1B) and *CYP3A5* (*1/*1 or *1/*3) (data not shown)*.* Carriers of the *MDR1 1236 C/C* genotype exhibited lower mean C_0_/D at week 1 compared to carriers of C/T (Figure 1D). No other genotype or genotype combination exhibited associations with C_0_/D or C_2_/D.

**FIGURE 3:**
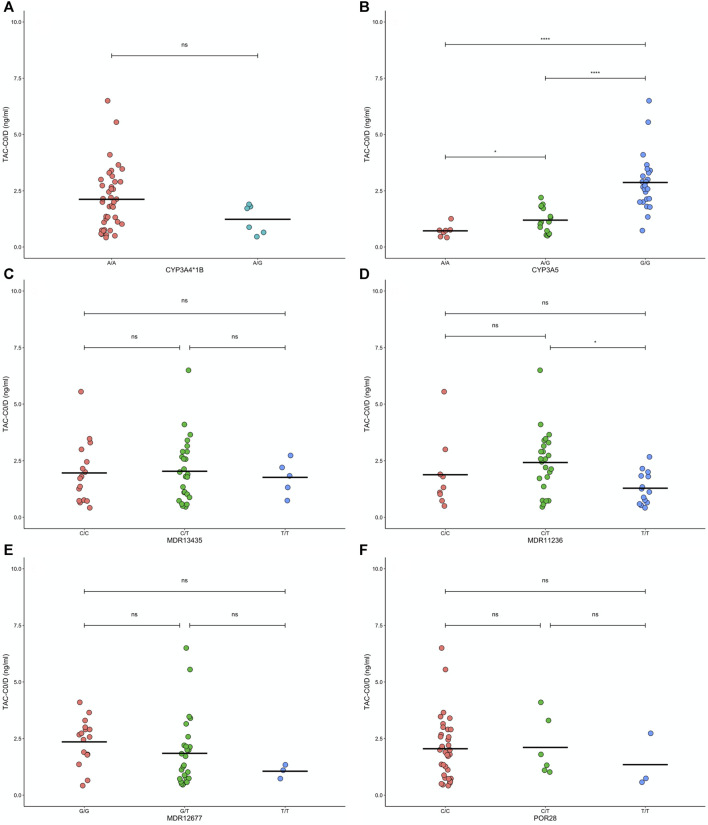
Levels of tacrolymus (TAC) normalized by dose before administration (TACC0/D) for 50 patients with different **(A)** CYP3A4*1B, **(B)** CYP3A5, **(C)** MDR1-3435, **(D)** MDR1-1236, **(E)** MDR1-2677, and **(F)** POR28 genotypes. Paired comparisons between genotypes using Bonferroni-adjusted t- tests are they results are indicated as nonsignificant (ns: *p* > 0.05) or significant (*p < 0.05, **p < 0.01, ***p < 0.001, and ****p < 0.0001).

For TAC treated patients, we tested associations between genotypes and C_0_/D 6 months to 14 years post-transplant (Figure 3). CYP3A5*3 A/A carriers and *A/G* carriers each exhibited lower mean C_0_/D levels compared to *G/G* carriers (Figure 3B, *p* < 0.05 for both). Carriers of *MDR1 1236 T/T* exhibited lower mean C_0_/D levels compared to *C/T* carriers (*p* = 0.012, Figure 3D). No other genotype exhibited associations with C_0_/D.

**FIGURE 4:**
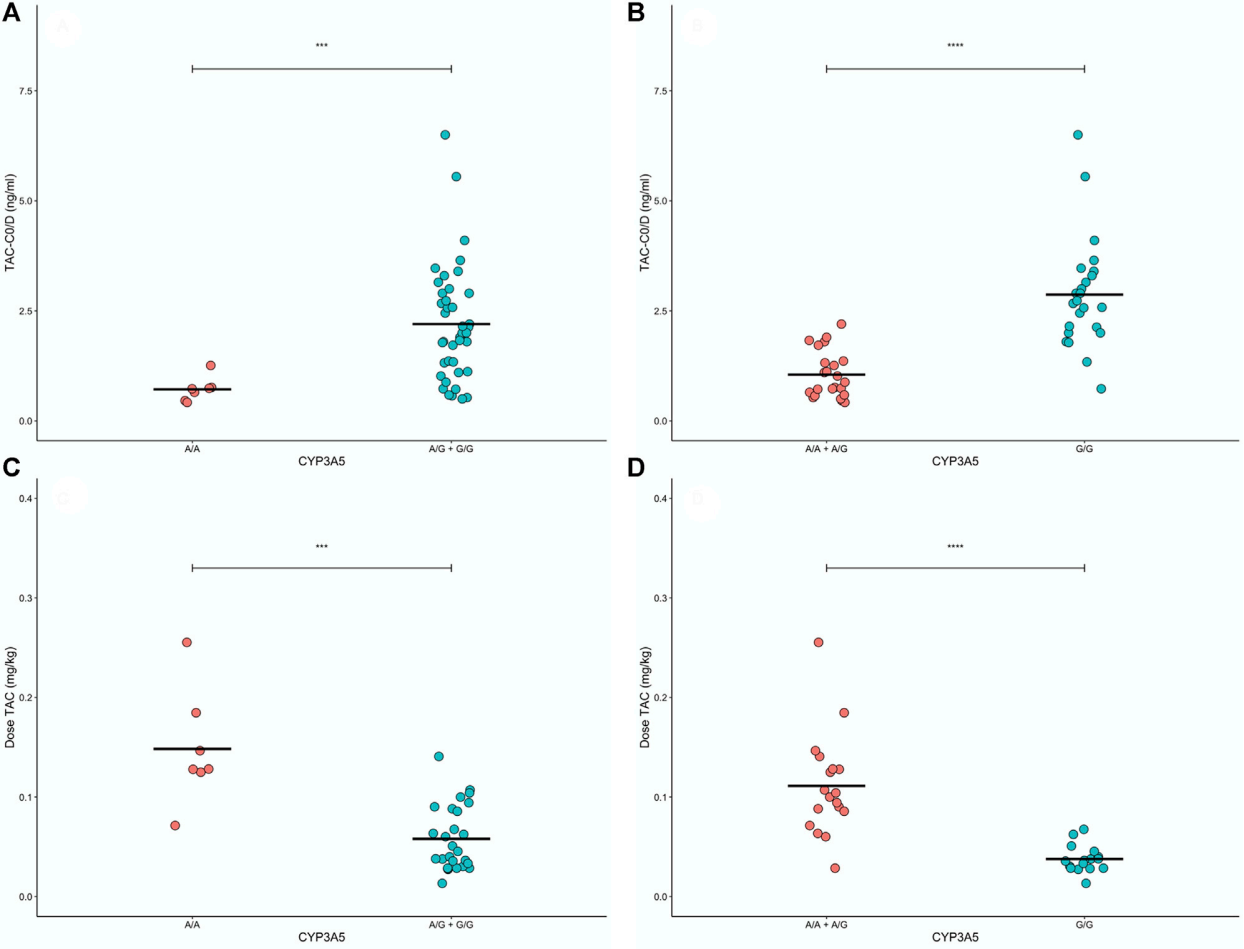
Levels of tacrolimus (TAC) normalized by dose before administration (TACC0/D) and tacrolimus dose standardized by patient weight according to dominant **(A,C)** and recessive **(B,D)** modes for CYP3A5 gene, respectively. ANOVA results are indicated as significant differences between grouped genotypes (***p < 0.001, and ****p < 0.0001).

We explored inheritance models for CYP3A5*3, *MDR1 1236* and *2677*. For CYP3A5*3 the strongest model suggested dominant inheritance, where *G/G* carriers exhibited 3-fold lower mean C0/D levels than *A/A* + *A/G* carriers (Figure 4). *MDR1 1236 C/C* + *C/T* carriers exhibited higher mean C_0_/D levels compared to *T/T* carriers, suggesting a recessive model (*p* = 0.026, Supplementary Figures S1A,B). *MDR1 2677 C/C* carriers exhibited higher mean C_0_/D compared to *C/T + T/T* carriers, suggesting a dominant mode (*p* = 0.017, Supplementary Figures S2A,B). The most common haplotype for *MDR1* is *TTT* (carriage of the polymorphic base T at *1236, 2677* and *3435*). We did not find a difference in mean C_0_/D between *MDR1 TTT* carriers and those with alternative haplotypes (Supplementary Figure S3).

### Genetic Association Between Polymorphisms and Dose Requirements

For CsA treated patients, we tested associations between genotypes and weight adjusted dose requirement at week 1, 2 and 4 and found no associations (Supplementary Figures S4A–G). For TAC treated patients, we found CYP3A5*3 A/A carriers had the highest weight normalized dose requirement, followed by A/G carriers and G/G carriers (ANOVA *p* < 0.05, Supplementary Figures S5,S6).

### Genetic Association Between Polymorphisms and Proportion of TAC Patients in Therapeutic Range

TAC treated patients were categorized as being inside or outside the therapeutic range at 6 months post-transplant (C_0_ 5–10 ng/ml), and we tested the influence of genotypes. CYP3A5*3 GG carriers were more frequently out of range compared to A/G and A/A carriers combined (*p* = 0.053, Table 3). CsA patients were not tested as they receive frequent drug monitoring in the period corresponding to the data available (within 1-month post-transplant).

**TABLE 3:** Proportion of patients in therapeutic range per genotype.

	CYP3A4*1B rs2740574–392A > G				CYP3A4*22 rs35599367 C191C		
	Wt (AA)	Het (AG)	*p*-value		Wt (CC)	Het (CT)	*p*-value
In range	23 (71.8%)	5 (83.3%)	1	In range	28 (68.3%)	2 (100%)	1
Out range	9 (28.2%)	1 (16.7%)		Out range	13 (31.7%)	0 (0%)	

	CYP3A5*3 rs776746 6986 A > G				CYP3A5*3 rs776746 6986 A > G		
	**Wt (AA)**	**Het (AG) + Mut (GG)**	***p*-value**		**Wt (AA) + Het (AG)**	**Mut (GG)**	***p*-value**
In range	5 (83.3%)	25 (67.6%)	0.649	In range	17 (85.0%)	13 (56.5%)	0.053
Out range	1 (16.7%)	12 (32.4%)		Out range	3 (15.0%)	10 (43.5%)	

	CYP3A5*3 rs776746 6986 A > G						
	Wt (AA)	Het (AG)	Mut (GG)	*p*-value			
In range	5 (83.3%)	12 (85.7%)	13 (56.5%)	0.168			
Out range	1 (16.6%)	2 (14.3%)	10 (43.5%)				

	POR*28 rs1057868 1508C > T				POR*28 rs1057868 1508C > T		
	Wt (CC)	Het (CT) + Mut (TT)	*p*-value		Wt (CC) + Het (CT)	Mut (TT)	*p*-value
In range	22 (66.7%)	8 (80%)	0.696	In range	27 (67.5%)	3 (100%)	0.5418
Out range	11 (33.3%)	2 (20%)		Out range	13 (32.5%)	0 (0%)	

	POR*28 rs1057868 1508C > T						
	Wt (CC)	Het (CT)	Mut (TT)	*p*-value			
In range	22 (66.7%)	5 (71.4%)	3 (100%)	0.841			
Out range	11 (33.3%)	2 (28.6%)	0 (0%)				

	MDR1 rs1045642 C3435T				MDR1 rs1045642 C3435T		
	Wt (CC)	Het (CT) + Mut (TT)	*p*-value		Wt (CC) + Het (CT)	Mut (TT)	*p*-value
In range	8 (61.5%)	22 (73.3%)	0.4854	In range	27 (69.2%)	3 (75%)	1
Out range	5 (38.5%)	8 (26.7%)		Out range	12 (30.8%)	1 (25%)	

	MDR1 rs1045642 C3435T						
	Wt (CC)	Het (CT)	Mut (TT)	*p*-value			
In range	8 (61.5%)	19 (73.1%)	3 (75.0%)	0.780			
Out range	5 (38.5%)	7 (26.9%)	1 (25.0%)				

	MDR1 rs1128503 C1236T				MDR1 rs1128503 C1236T		
	Wt (CC)	Het (CT) + Mut (TT)	*p*-value		Wt (CC) + Het (CT)	Mut (TT)	*p*-value
In range	6 (75%)	24 (68.6%)	1 (%)	In range	21 (67.7%)	9 (75%)	0.7272
Out range	2 (25%)	11 (31.4%)		Out range	10 (32.3%)	3 (25%)	

	MDR1 rs1128503 C1236T						
	Wt (CC)	Het (CT)	Mut (TT)	*p*-value			
In range	6 (75%)	15 (65.2%)	9 (75%)	0.907			
Out range	2 (25%)	8 (34.8%)	3 (25%)				

	MDR1 2677 rs2032582 C2677T/A				MDR1 2677 rs2032582 G2677T		
	Wt (CC)	Het (CT) + Mut (TT)	*p*-value		Wt (CC) + Het (CT)	Mut (TT)	*p*-value
In range	12 (66.7%)	18 (72.0%)	0.7466	In range	29 (70.7%)	1 (50%)	0.5183
Out range	6 (33.3%)	7 (28.0%)		Out range	12 (29.2%)	1 (50%)	

	MDR1 2677 rs2032582 G2677T						
	Wt (CC)	Het (CT)	Mut (TT)	*p*-value			
In range	12 (66.7%)	17 (73.9%)	1 (50%)	0.646			
Out range	6 (33.3%)	6 (26.1%)	1 (50%)				

	MDR1 TTT haplotype	MDR1 non-TTT haplotype	*p*-value				
In range	10 (66.7%)	20 (71.4%)	0.7422			
Out range	5 (33.3%)	8 (28.6%)	

*p*-value: Fisher’exact test.

## Discussion

Our aim was to test associations between genetic polymorphisms involved in the absorption and metabolism of CsA and TAC, and drug disposition variables in two groups of Chilean kidney transplant patients, adding to an existing body of work performed in predominantly Caucasian, Asian and African American populations (Staatz et al., 2010). Consistent with existing work, we found CYP3A5*3/*3 (G/G) carriers required lower doses of TAC, presented higher C_0_/D and a higher proportion of patients with C_0_ levels outside the therapeutic range relative to other genotypes (Birdwell et al., 2015). Of note, in the TAC group the A allele is more frequent in patients within the therapeutic range, possibly due to a more stable drug disposition when at least one functional allele is expressed. As expected, no significant associations were found for this allele in the CsA cohort, likely due to a lower affinity of CYP3A5 for this drug.

We found *MDR1* 2677 C/C associated with higher TAC trough levels. This is consistent with *MDR1-*2677 C resulting in decreased protein expression and causing reduced drug efflux. *MDR1* 1236 T/T associated with lower TAC levels and higher doses, although the functional consequence of this variant is unclear and divergent results have been published (Staatz et al., 2010). The *MDR1 TTT* haplotype, combining polymorphic variants at positions 1236, 2677 and 3435, did not associate with C_0_ or TAC dose. We therefore conclude *MDR1* variants do not significantly affect drug disposition, or may have a minor impact likely to be clinically irrelevant (Phuthong et al., 2017; Robertsen et al., 2018).

Our work has a number of limitations. Firstly, both cohorts received limited follow-up. Further, we did not take into account multi-drug immunosuppressive regimens that may have varied between patients (secondary immunosuppressant and/or corticosteroid). Thirdly, we did not perform a sample size calculation as for a Latin American population the effect size for the impact of each polymorphism on the variables studied, and for each immunosuppressant studied, is uncertain, and the unpredictable frequency of kidney transplants at both hospitals during the enrolment period.

The *CYP3A5* genotyping to guide a tacrolimus starting dose is a clinically relevant practice that is currently undertaken in several centers around the world. It may indeed prove a cost-effective addition to the clinical tool kit to ensure that patients, that already receive therapeutic drug monitoring, achieve levels within the therapeutic range faster. Further, it could flag patients that require closer monitoring to ensure levels within the therapeutic range at 6 months post-transplant and beyond, such as those out of range in our cohort. The ultimate aim is to ensure patients receive adequate immunosuppression, keep their graft patent and avoid adverse effects related to overexposure. Data on cost effectiveness and important clinical endpoints are deficient, especially for lower/middle income countries, including those in Latin America. Further work on these aspects is warranted.

## Data Availability

Raw data can be accessed here: https://figshare.com/articles/dataset/Datasets/14128547/1

## Supplementary Material

Supplementary Figure S1 C0 of TAC patients associated to MDR1 1236 polymorphism. **(A)** Co-dominant model. **(B)** Dominant model. **(C)** Recessive model. Significance level: *p*-value < 0.05. See genetic polymorphisms details in Supplementary Table S1.

Supplementary Figure S2 C0 of TAC patients associated to MDR1 2677 polymorphism. **(A)** Co-dominant model. **(B)** Dominant model. **(C)** Recessive model. Significance level: *p*-value < 0.05. See genetic polymorphisms details in Supplementary Table S1.

Supplementary Figure S3 C0 of TAC patients associated to MDR1 TTT haplotype. MDR1 haplotype configured by C3435T, C1236T and C2677T/A. Significance level: *p*-value < 0.05. See genetic polymorphisms details in Supplementary Table S1.

Supplementary Figure S4 TAC-Dose associations to CYP3A5 polymorphism. **(A)** Co-dominant model. **(B)** Dominant model. **(C)** Recessive model. TAC: tacrolimus. Significance level: *p*-value < 0.05. See genetic polymorphisms details in Supplementary Table S1.

Supplementary Figure S5 CsA Dose/weight per genotype. Repeated-measured ANOVA. W1: week 1. W2: week 2. W4: week 4. Significance level: *p*-value < 0.05. See genetic polymorphisms details in Supplementary Table S1.

Supplementary Figure S6 TAC Dose/weight per genotype. ANOVA test and T test. Significance level: *p*-value < 0.05. See genetic polymorphisms details in Supplementary Table S1.

Supplementary Figure S7 TAC-Dose associated to MDR1 TTT haplotype. MDR1 haplotype configured by C3435T, C1236T, and C2677T/A. Significance level: *p*-value < 0.05. See genetic polymorphisms details in Supplementary Table S1.

## References

[B1] Amirimani B. Ning B. Deitz A. C. Weber B. L. Kadlubar F. F. Rebbeck T. R. (2003). Increased Transcriptional Activity of the CYP3A4*1B Promoter Variant. Environ. Mol. Mutagen 42 (4), 299–305. 10.1002/em.10199 14673875

[B2] Bikbov B. Purcell C. A. Levey A. S. Smith M. Abdoli A. Abebe M. (2020). Global, Regional, and National Burden of Chronic Kidney Disease, 1990–2017: A Systematic Analysis for the Global Burden of Disease Study 2017. The Lancet 395 (10225), 709–733. 10.1016/S0140-6736(20)30045-3 PMC7049905 32061315

[B3] Birdwell K. A. Decker B. Barbarino J. M. Peterson J. F. Stein C. M. Sadee W. (2015). Clinical Pharmacogenetics Implementation Consortium (CPIC) Guidelines for CYP3A5 Genotype and Tacrolimus Dosing. Clin. Pharmacol. Ther. 98 (1), 19–24. 10.1002/cpt.113 25801146 PMC4481158

[B4] Cotovio P. Neves M. Rodrigues L. Alves R. Bastos M. Baptista C. (2013). New-Onset Diabetes after Transplantation: Assessment of Risk Factors and Clinical Outcomes. Transpl. Proc 45 (3), 1079–1083. 10.1016/j.transproceed.2013.03.009 23622631

[B5] Egeland E. J. Robertsen I. Hermann M. Midtvedt K. Størset E. Gustavsen M. T. (2017). High Tacrolimus Clearance Is a Risk Factor for Acute Rejection in the Early Phase after Renal Transplantation. Transplantation 101 (8), e273–79. 10.1097/TP.0000000000001796 28452920

[B6] Elens L. Nieuweboer A. J. Clarke S. J. Charles K. A. de Graan A. J. Haufroid V. (2013). Impact of POR*28 on the Clinical Pharmacokinetics of CYP3A Phenotyping Probes Midazolam and Erythromycin. Pharmacogenet Genomics 23 (3), 148–155. 10.1097/FPC.0b013e32835dc113 23324807

[B7] Gelder T. V. Meziyerh S. Swen J. J. Aiko P. de Vries J. Moes D. J. A. R. (2020). The Clinical Impact of the C0/D Ratio and the CYP3A5 Genotype on Outcome in Tacrolimus Treated Kidney Transplant Recipients. Front. Pharmacol. 11, 1–6. 10.3390/genes11101205 32848756 PMC7411304

[B8] Hesselink D. A. Bouamar R. Elens L. van SchaikVan Schaik R. H. van Gelder T. (2014). The Role of Pharmacogenetics in the Disposition of and Response to Tacrolimus in Solid Organ Transplantation. Clin. Pharmacokinet. 53 (2), 123–139. 10.1007/s40262-013-0120-3 24249597

[B9] Jiang Z. P. Wang Y. R. Xu P. Liu R. R. Zhao X. L. Chen F. P. (2008). Meta-Analysis of the Effect of MDR1 C3435T Polymorphism on Cyclosporine Pharmacokinetics. Basic Clin. Pharmacol. Toxicol. 103 (5), 433–444. 10.1111/j.1742-7843.2008.00300.x 18801030

[B10] Jonge H. D. Metalidis C. Naesens M. Lambrechts D. Dirk R. Kuypers J. (2011). The P450 Oxidoreductase *28 SNP Is Associated with Low Initial Tacrolimus Exposure and Increased Dose Requirements in CYP3A5-Expressing Renal Recipients. Pharmacogenomics 12 (9), 1281–1291. 10.2217/pgs.11.77 21770725

[B11] Kuehl P. Zhang J. Lin Y. Lamba J. Assem M. Schuetz J. (2001). Sequence Diversity in CYP3A Promoters and Characterization of the Genetic Basis of Polymorphic CYP3A5 Expression. Nat. Genet. 27 (4), 383–391. 10.1038/86882 11279519

[B12] Okubo M. Murayama N. Shimizu M. Shimada T. Guengerich F. P. Yamazaki H. (2013). CYP3A4 Intron 6 C>T Polymorphism (CYP3A4*22) Is Associated with Reduced CYP3A4 Protein Level and Function in Human Liver Microsomes. J. Toxicol. Sci. 38 (3), 349–354. 10.2131/jts.38.349 23665933 PMC4018728

[B13] Phuthong S. Settheetham-Ishida W. Natphopsuk S. Settheetham D. Ishida T. (2017). Haplotype Analysis of MDR1 and Risk for Cervical Cancer in Northeastern Thailand. Asian Pac. J. Cancer Prev. 18 (7), 1815–1819. 10.22034/APJCP.2017.18.7.1815 28749110 PMC5648384

[B14] Robertsen I. Debord J. Åsberg A. Marquet P. Woillard J. B. (2018). A Limited Sampling Strategy to Estimate Exposure of Everolimus in Whole Blood and Peripheral Blood Mononuclear Cells in Renal Transplant Recipients Using Population Pharmacokinetic Modeling and Bayesian Estimators. Clin. Pharmacokinet. 57, 1459–1469. 10.1007/s40262-018-0646-5 29556934

[B15] Rodrigo E. Segundo D. S. Fernández-Fresnedo G. López-Hoyos M. Benito A. Ruiz J. C. (2016). Within-Patient Variability in Tacrolimus Blood Levels Predicts Kidney Graft Loss and Donor-Specific Antibody Development. Transplantation 100 (11), 2479–2485. 10.1097/TP.0000000000001040 26703349

[B16] Staatz C. E. Goodman L. K. Tett S. E. (2010). Effect of CYP3A and ABCB1 Single Nucleotide Polymorphisms on the Pharmacokinetics and Pharmacodynamics of Calcineurin Inhibitors: Part II. Clin. Pharmacokinet. 49 (3), 207–221. 10.2165/11317550-000000000-00000 20214406

[B17] Sun B. Guo Y. Gao J. Shi W. Fan G. Li X. (2017). Influence of CYP3A and ABCB1 Polymorphisms on Cyclosporine Concentrations in Renal Transplant Recipients. Pharmacogenomics 18 (16), 1503–1513. 10.2217/pgs-2017-0127 28952408

[B18] Traynor C. Conlon P. Phelan P. J. O'Kelly P. Elens L. Mccormack M. (2015). Association of CYP3A Variants with Kidney Transplant Outcomes. Ren. Fail. 37 (4), 562–566. 10.3109/0886022X.2015.1007013 25644970 PMC13580358

[B19] Webster A. C. Woodroffe R. C. Taylor R. S. Chapman J. R. Craig J. C. (2005). Tacrolimus Versus Ciclosporin as Primary Immunosuppression for Kidney Transplant Recipients: Meta-Analysis and Meta-Regression of Randomised Trial Data. BMJ 331 (7520), 810. 10.1136/bmj.38569.471007.AE 16157605 PMC1246079

