# Supplementary figures and images for: Effect of CYP3A4, CYP3A5, MDR1 and POR Genetic Polymorphisms in Immunosuppressive Treatment in Chilean Kidney Transplanted Patients
Source: Front Pharmacol. 2021 Dec 6;12:674117. doi: 10.3389/fphar.2021.674117 (PMC8685429; doi:10.3389/fphar.2021.674117)

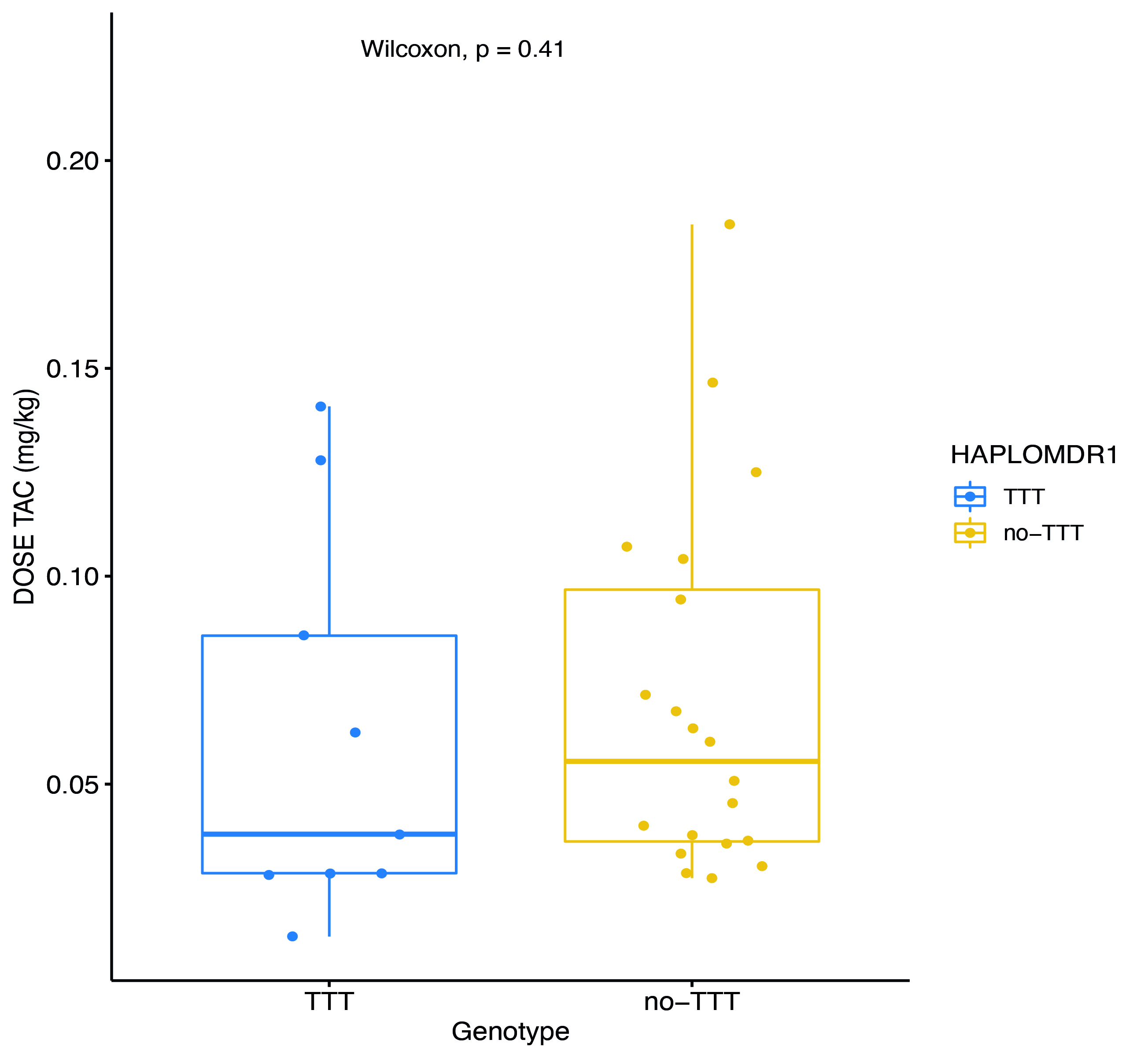

Supplement: Supplementary file 1 [file Image3.jpeg]

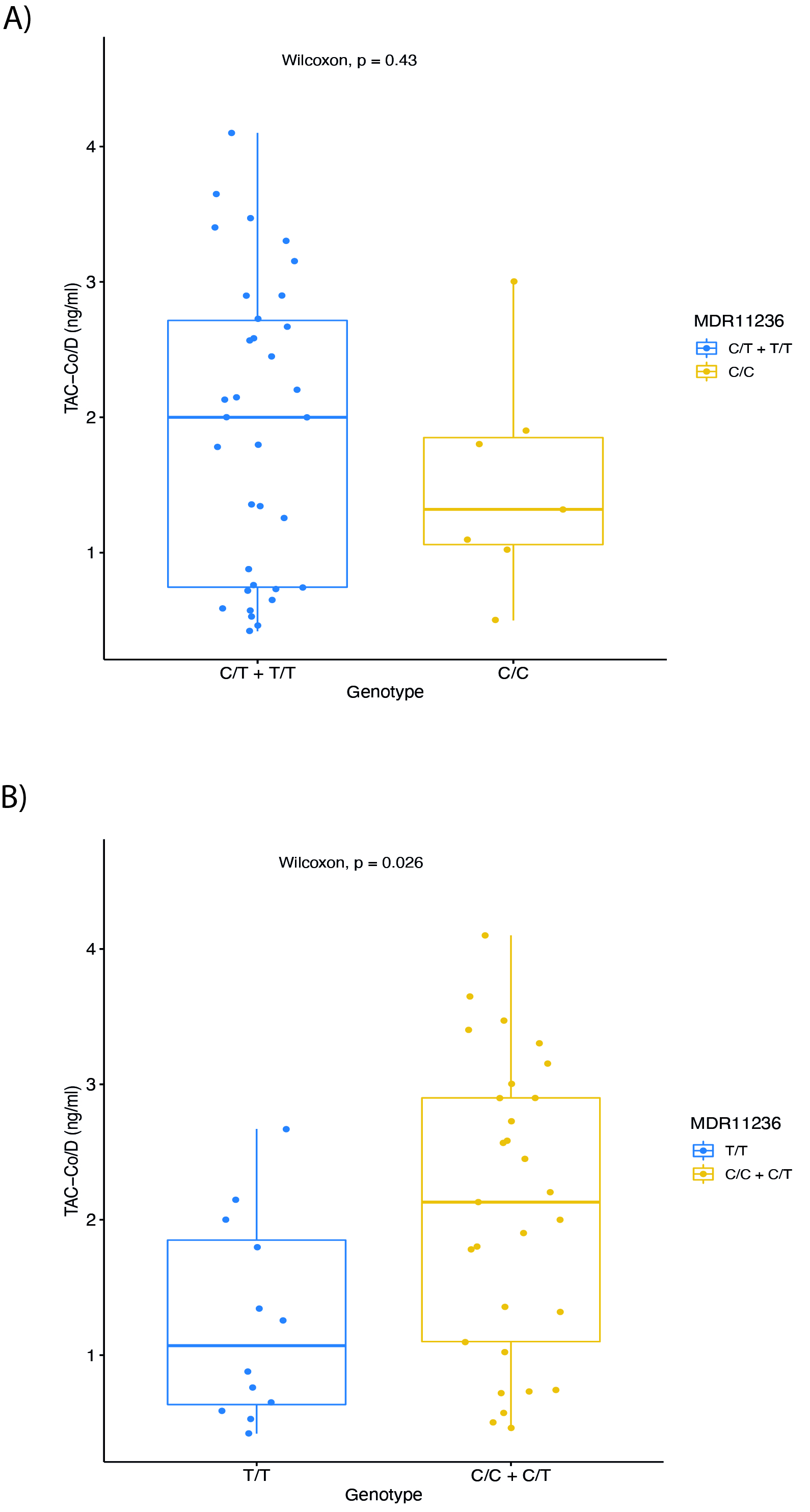

Supplement: Supplementary file 2 [file Image1.JPEG]

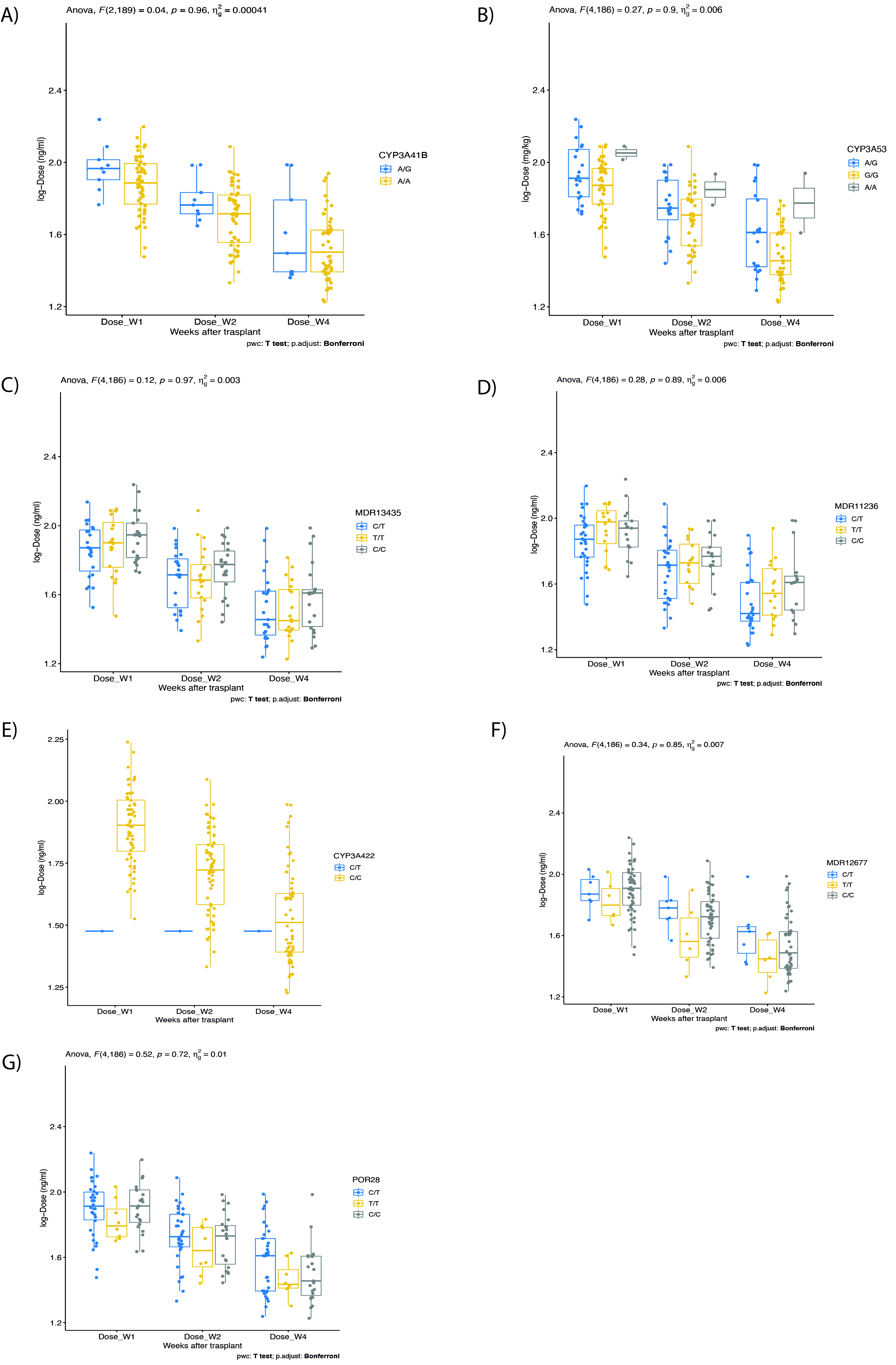

Supplement: Supplementary file 3 [file Image4.JPEG]

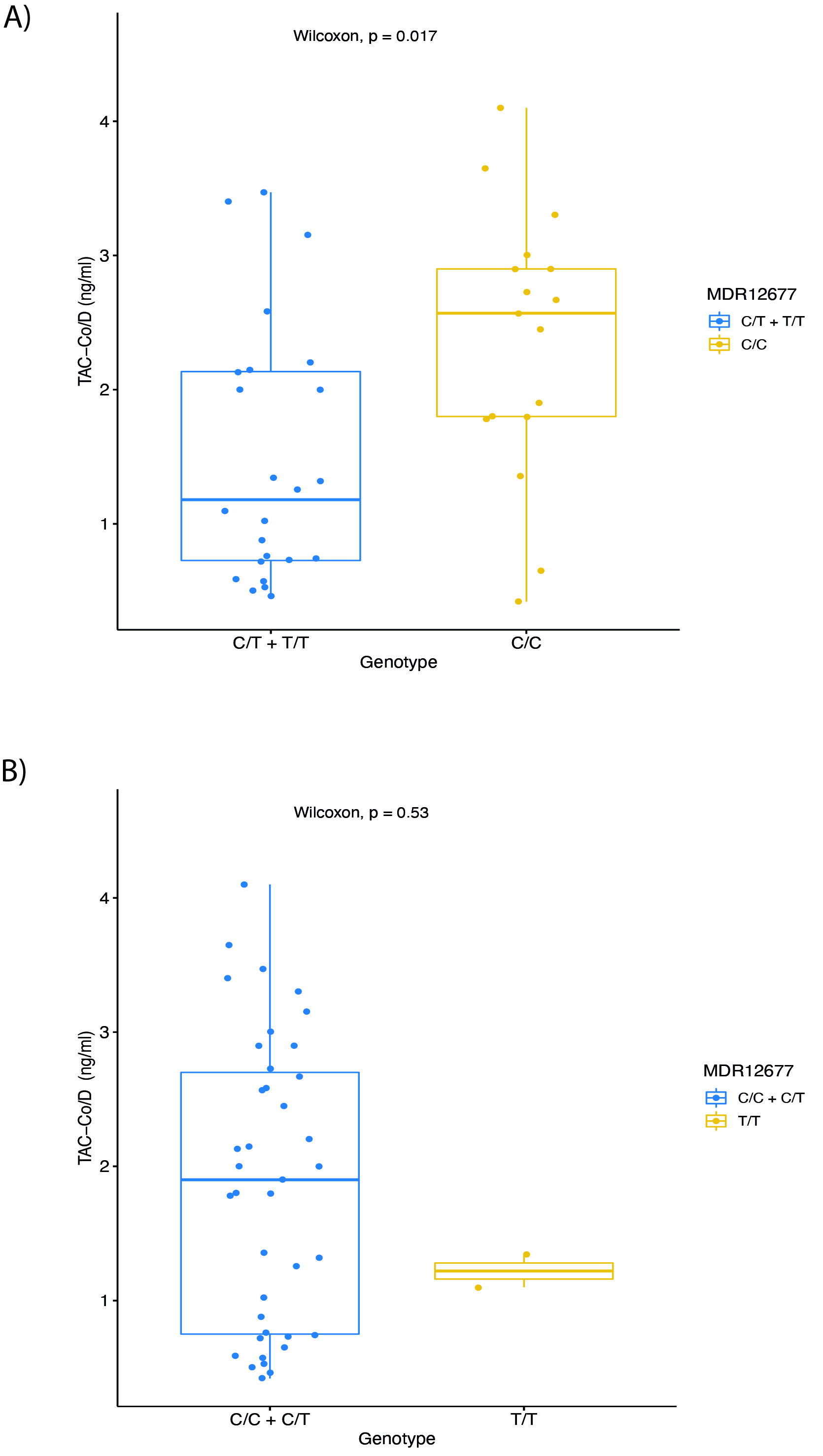

Supplement: Supplementary file 5 [file Image2.JPEG]

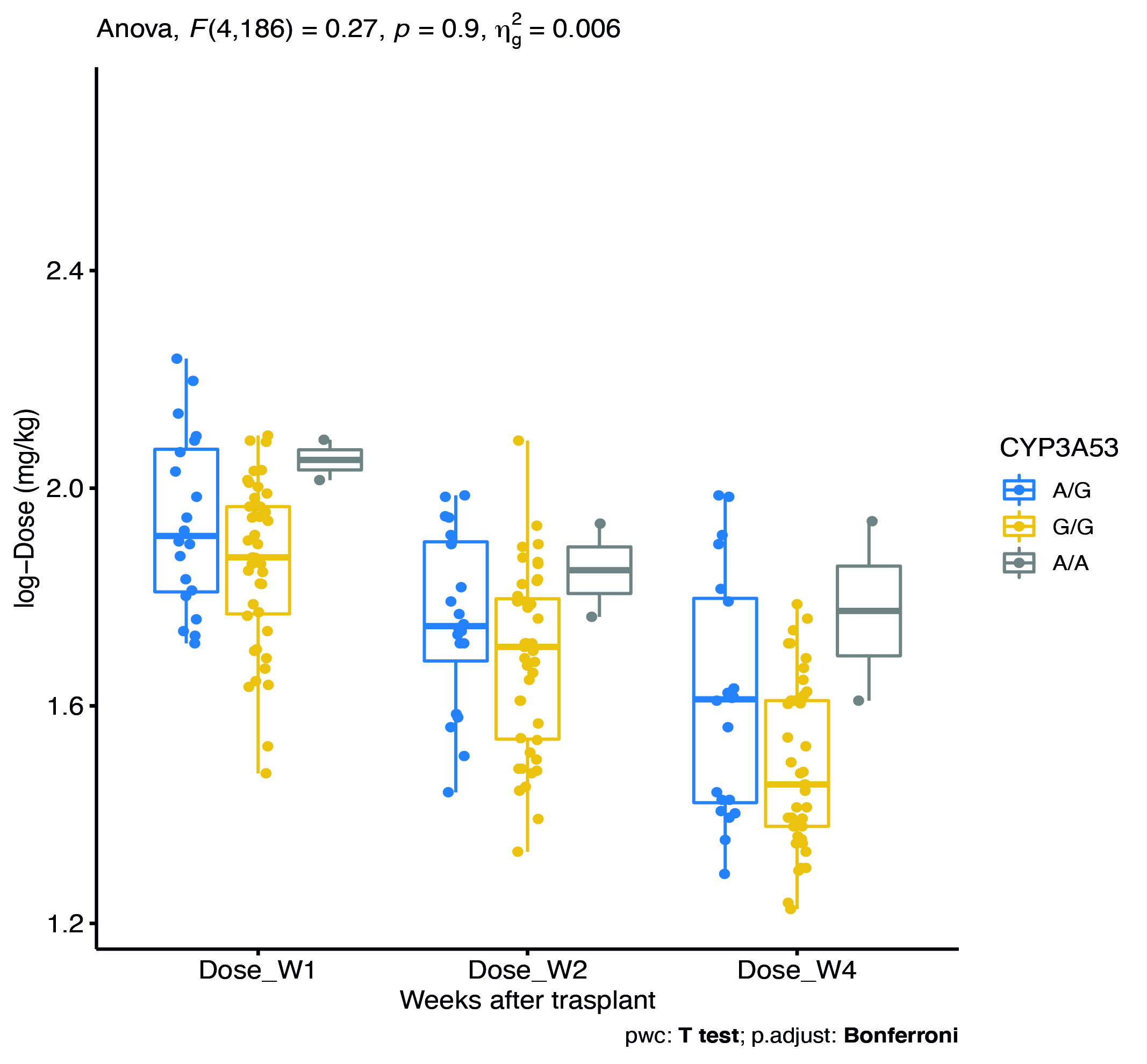

Supplement: Supplementary file 6 [file Image5.JPEG]

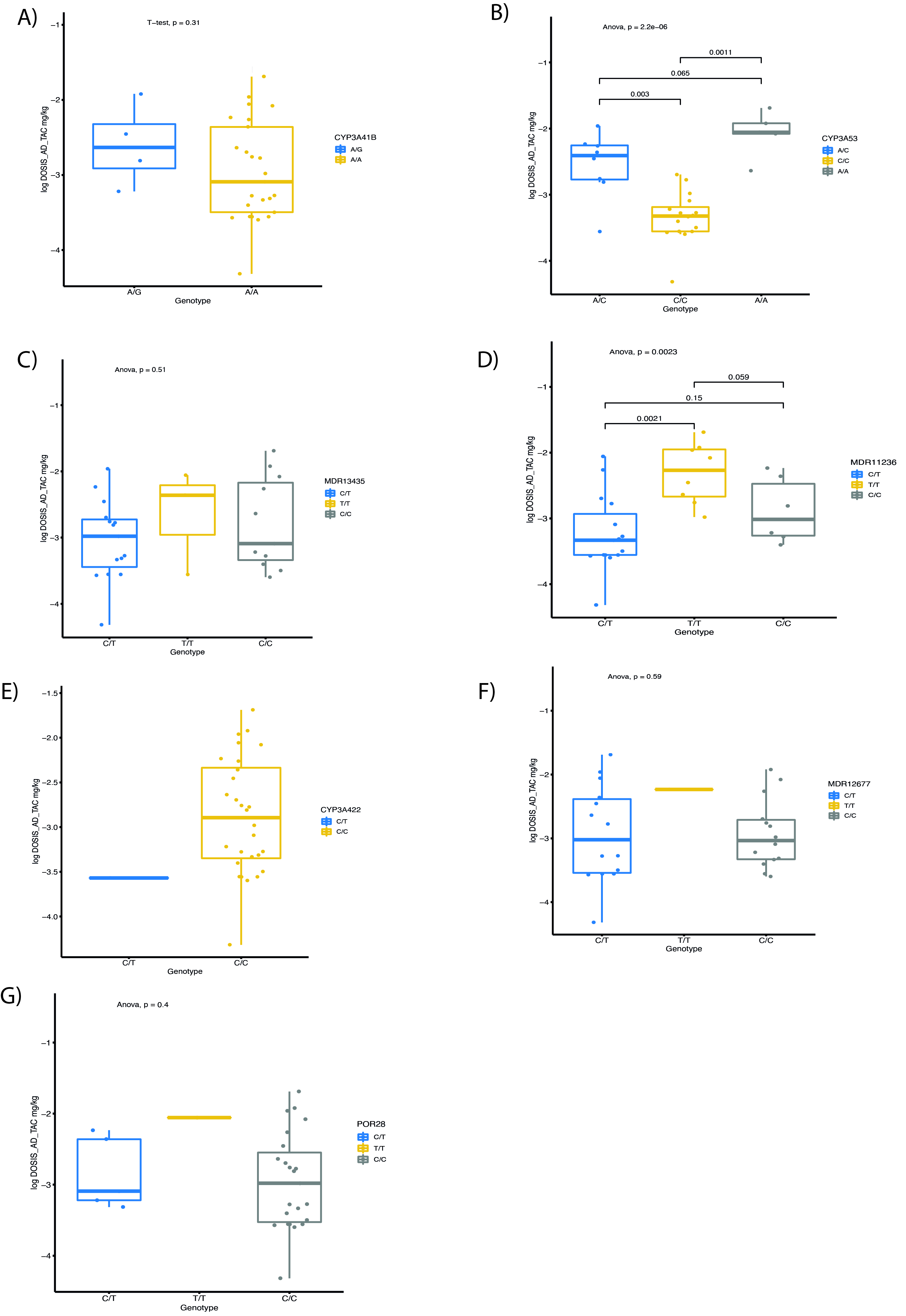

Supplement: Supplementary file 7 [file Image6.JPEG]
